# Soft tissue laxity is highly variable in patients undergoing total knee arthroplasty

**DOI:** 10.1186/s42836-024-00268-w

**Published:** 2024-08-07

**Authors:** Travis R. Weiner, Roshan P. Shah, Alexander L. Neuwirth, Jeffrey A. Geller, H. John Cooper

**Affiliations:** Department of Orthopedic Surgery, Columbia University Medical Center, New York, NY 10032 USA

**Keywords:** Total knee arthroplasty, Robotic-assisted surgery, Soft tissue laxity, Gap-balancing

## Abstract

**Background:**

One major goal of total knee arthroplasty (TKA) is to achieve balanced medial and lateral gaps in flexion and extension. While bone resections are planned by the surgeon, soft tissue laxity is largely intrinsic and patient-specific in the absence of additional soft tissue releases. We sought to determine the variability in soft tissue laxity in patients undergoing TKA.

**Methods:**

We retrospectively reviewed 113 patients undergoing TKA. Data on preoperative knee deformity were collected. Data from a dynamic intraoperative stress examination were collected by a robotic tracking system to quantify maximal medial and lateral opening in flexion (85–95 degrees) and extension (-5–20 degrees). T-tests were used to assess the differences between continuous variables.

**Results:**

A valgus stress opened the medial compartment a mean of 4.3 ± 2.3 mm (0.0–12.4 mm) in extension and 4.6 ± 2.3 mm (0.0–12.9 mm) in flexion. A varus stress opened the lateral compartment a mean of 5.4 ± 2.4 mm (0.3–12.6 mm) in extension and 6.2 ± 2.5 mm (0.0–13.4 mm) in flexion.

The medial compartment of varus knees opened significantly more in response to valgus stress than valgus knees in both extension (5.2 mm vs. 2.6 mm; *P* < 0.0001) and flexion (5.4 mm vs 3.3 mm; *P* < 0.0001). The lateral compartment of valgus knees opened significantly more in response to varus stress than varus knees in both extension (6.7 mm vs. 4.8 mm; *P* < 0.0001) and flexion (7.4 mm vs. 5.8 mm; *P* = 0.0003).

**Conclusions:**

Soft tissue laxity is highly variable in patients undergoing TKA, contributing anywhere from 0–13 mm to the post-resection gap. Only a small part of this variability is predictable by preoperative deformity. These findings have implications for either measured-resection or gap-balancing techniques.

**Level of Evidence:**

Level III.

## Introduction

One major goal of total knee arthroplasty (TKA) is to achieve balanced gaps in medial and lateral compartments in both flexion and extension. Gap-balancing has been theorized to be integral to the overall success of TKA and is an important factor for postoperative function, implant durability, and patient satisfaction [1–3]. Poor balance may lead to instability, pain, increased polyethylene wear, aseptic loosening, and ultimately may result in revision TKA [4–7].

Flexion and extension gaps are the sum of (a) the thickness of bone cuts and (b) the soft tissue laxity in each compartment. Soft tissue laxity itself is not just the ligamentous laxity but also the correctability of the deformity. Surgical experience tells us some arthritic knees develop soft tissue contractures on the diseased side that are fixed or minimally correctable, while others exhibit much more correctability, with laxity that may be greater than anticipated in the diseased compartment if there is significant bone loss in the setting of a correctable deformity. This idea of cartilage degeneration and bone loss potentially leading to increased perceived laxity in the diseased compartment is commonly referred to as “pseudolaxity” and can be attributed to a decrease in distance between ligament attachment points [8]. It is important to consider this “pseudolaxity” as a part of the overall soft tissue laxity as it will likely affect bone resections and ultimately the ability to achieve balanced gaps.

Various surgical strategies are available to achieve balanced gaps [9], each having potential advantages and limitations. While the thickness of the bone cuts is fully within the surgeon’s control during the procedure, the pre-existing soft tissue laxity is largely patient-specific and can be highly variable. Determining the ideal amount of bone to resect in each compartment can be difficult without knowledge of the corresponding soft tissue laxity in that compartment when a measured resection approach is considered a surgical principle while performing TKA surgery.

With that variability in mind, prior literature has shown that the ideal amount of bone resection and soft tissue release cannot be predicted based on preoperative deformity [10]. With the progression of coronal plane deformity, it is often unclear when there is progressive soft tissue contracture or increased laxity of the medial and lateral soft tissues. Prior studies have reported variability in the medial soft tissue contracture or laxity in knees with a preoperative varus deformity and that medial soft tissue contracture is not universal [11, 12]. While bone resections can be planned by the surgeon, these data suggest that soft tissue laxity in varus knees can vary, is largely intrinsic, and is patient-specific in the absence of additional soft tissue releases. While several studies described medial and lateral soft tissue laxity based on varus deformity, it remains unclear how variable soft tissue laxity can be in patients with a valgus deformity, and how that variability compares to that in their counterparts with varus deformity.

The purpose of this study was to determine the variability in both medial and lateral soft tissue laxity in patients undergoing TKA. Our study hypothesis was that both medial and lateral soft tissue laxity are highly variable between patients and cannot be accurately predicted by preoperative deformity.

## Materials and methods

After obtaining approval from our institutional review board, a retrospective analysis of consecutive primary TKA cases performed by two fellowship-trained arthroplasty surgeons between September 2020 and May 2022 was completed. The preoperative knee deformity as well as patient demographics, including sex and age, were documented.

Data from a dynamic intraoperative manual stress exam were collected using a robotic knee system (ROSA® Knee System, Zimmer, Inc, Warsaw, IN, USA) to quantify maximal laxity in medial and lateral compartments. Both surgeons had significant experience and were well past the initial learning curve for the robotic knee system that was used. Cases that did not have complete robotic data were excluded from this analysis.

The manual stress exam captures the maximal distraction of the femoral condyle and the tibial plateau during the dynamic stress application and has been shown to have a high intra- and inter-rater reliability [13]. Using the guidelines of the robotic system, maximal compartment laxity was acquired dynamically in both flexion (85 to 95 degrees) and extension (-5 to 20 degrees). These parameters were defined by the robotic system used for data acquisition and allowed the maximum laxity over that defined range of motion to be captured in each compartment during the dynamic surgeon-applied stress exam. The allowed extension range was greater to account for knees that had flexion contractures and could not reach full extension. This stress exam was performed after exposure, resection of the anterior cruciate ligament, resection of the posterior cruciate ligament when sacrificed by surgeon preference, removal of accessible osteophytes, and standard registration of landmarks. The stress exam was performed before any bony resections or soft tissue releases beyond a standard surgical exposure. The robot measured the resultant gaps (in millimeters), which is the sum of the gaps plus any bone defects, of the medial side with valgus stress and similarly of the lateral side with varus stress.

### Statistical analysis

Preoperative knee deformity was examined to determine the variability in medial and lateral soft tissue laxity in patients undergoing TKA, and how this variability may differ based on preoperative knee deformity. Statistical analyses were done with SPSS (version 28.0, SPSS Inc., Chicago, IL, USA). *t*-tests were conducted for continuous variables, to compare valgus and varus knees based on the size of the stressed gaps. All *P* values were for 2-sided tests, and a *P* value < 0.05 was considered statistically significant. A sample size calculation showed that to achieve 80% statistical power (β = 0.20) with α = 0.05 and a 2:1 enrollment ratio, a minimum of 21 total subjects was required to distinguish differences between groups.

## Results

A total of 113 primary TKAs with complete robotic data were performed in 99 patients. The mean patient age was 67.2 years (range, 41 to 88) and 66 of the 99 patients (66.6%) were women. Of the 113 knees, 71 (62.8%) had a preoperative varus deformity, 36 (31.9%) had a preoperative valgus deformity, and 6 (5.3%) had a neutral preoperative alignment.

A valgus stress applied to all knees opened the medial compartment a mean of 4.3 ± 2.3 mm (0.0–12.4 mm) in extension and 4.6 ± 2.3 mm (0.0–12.9 mm) in flexion. A varus stress opened the lateral compartment a mean of 5.4 ± 2.4 mm (0.3–12.6 mm) in extension and 6.2 ± 2.5 mm (0.0–13.4 mm) in flexion (Table 1).

**Table 1:** Results of medial and lateral compartment opening when varus and valgus stress were applied to all knees in extension and flexion

All patients (*n* = 113)	Varus stress/lateral gap compartment opening	Valgus stress/medial compartment opening
Extension	5.4 ± 2.4 mm (0.3–12.6 mm)	4.3 ± 2.3 mm (0.0–12.4 mm)
Flexion	6.2 ± 2.5 mm (0.0–13.4 mm)	4.6 ± 2.3 mm (0.0–12.9 mm)

The medial compartment of varus knees opened significantly more to valgus stress than in valgus knees in both extensions (5.2 mm vs 2.6 mm; *P* < 0.0001) and flexion (5.4 mm vs 3.3 mm; *P* < 0.0001) (Table 2). The lateral compartment of valgus knees opened significantly more to varus stress than in varus knees in both extension (6.7 mm vs 4.8 mm; *P* < 0.0001) and flexion (7.4 mm vs 5.8 mm; *P* = 0.0003) (Table 3).

**Table 2:** Results of t-test analysis of medial compartment opening when valgus stress was applied to both varus and valgus knees in extension and flexion

	Varus Knees (*n* = 71)	Valgus Knees (*n* = 36)	*P*
Extension	5.2 mm ± 2.1 mm (1.4–12.4 mm)	2.6 mm ± 1.6 mm (0–6.5 mm)	< 0.0001
Flexion	5.4 mm ± 2.3 mm (0–12.9 mm)	3.3 mm ± 1.7 mm (0.5–7.9 mm)	< 0.0001

**Table 3:** Results of t-test analysis of lateral compartment opening when varus stress was applied to both varus and valgus knees in extension and flexion

	Varus Knees (*n* = 71)	Valgus Knees (*n* = 36)	*P*
Extension	4.8 mm ± 2.2 mm (0.5–8.7 mm)	6.7 mm ± 2.2 mm (2.7–12.6 mm)	< 0.0001
Flexion	5.8 mm ± 2.4 mm (0–12.6 mm)	7.4 mm ± 2.6 mm (2.6–13.4 mm)	0.0003

## Discussion

Unbalanced gaps after TKA are undesirable and may lead to failure of the components and decreased postoperative function and patient satisfaction [1–3]. The most important findings of the present study were that compartment laxity, with components of both soft tissue laxity and the correctability of the deformity through pseudolaxity, is highly variable in patients undergoing TKA, contributing anywhere from 0–13 mm to the post-resection gap in each compartment. These findings are universally important for TKA surgery, regardless of whether performed with conventional instrumentation or with technology such as robotics. Newer studies by Graichen et al. [14] and Eller et al. [15] similarly showed that there was large variability in both extension and flexion gaps between patients’ varus and valgus knees. However, the findings in the current study, where varus knees opened more in the medial compartment than valgus knees and valgus knees opened more in the lateral compartment than varus knees, were contrasted by the results of Graichen et al. [14] and Eller et al. [15] who found that varus knees have larger lateral gaps and valgus knees have larger medial gaps. These contrasting findings might relate to differences in the method used for measuring resultant gaps and soft tissue laxity.

These findings have implications for either measured-resection or gap-balancing techniques. For example, with a measured resection technique where the surgeon aims to resect a fixed amount of bone from anatomic reference points, the post-resection gap in that compartment may vary among patients up to 13 mm based on their intrinsic soft tissue contractures or laxity. To achieve a balanced knee, this gap variability would then need to be addressed with subsequent techniques such as soft tissue releases or reduction osteotomies. In contrast, with a gap-balancing technique where the surgeon aims to cut the bone to achieve a balanced gap and often aided by a ligament tensioning device, they should expect the amount of bone resected from the relevant compartment to vary by as much as 13 mm among patients. This highlights the importance of considering “pseudolaxity” when measuring overall soft tissue laxity as the amount of cartilage degeneration and bone loss may contribute to a larger gap in the diseased compartment in the presence of a correctable deformity, although this larger gap may subsequently reduce the amount of bone resection that is necessary to achieve balanced gaps. The effects of these variable bony resections on the coronal, sagittal, and axial alignment of components may not be fully understood.

A number of studies examined soft tissue laxity in patients with a varus knee deformity undergoing TKA. Consistent with our results, Ushio et al. [11] found that there was not always a contracture of the medial soft tissues in patients with varus deformity and that any contracture, if present, is variable and patient-specific. Those authors concluded that underestimating the medial joint laxity and overestimating the implications of lateral soft tissue laxity in patients with varus knees may result in excessive medial tissue release and increase the risks of both postoperative instability and reduced patient satisfaction [10]. In contrast, Bellemans et al. [16] observed a consistent contraction of medial soft tissues in varus knees with preoperative deformity greater than 10 degrees. Additionally, there is a general agreement that there is some stretching of the lateral soft tissues in patients with a varus deformity [12, 16, 17]. Even with this agreement, Toyooka et al. [17] suggested that lateral soft tissue laxity is negligible as it is largely addressed by proper liner thickness, and Sekiya et al. [18] reported that lateral soft tissue laxity diminished with time after TKA.

In contrast to a multitude of studies on patients with varus knees, few studies examined soft tissue laxity in valgus deformity. On the basis of our findings, we are led to conclude that, like the medial soft tissue laxity in patients with varus deformity, the lateral soft tissue laxity in patients with valgus deformity is also quite variable and not predictable based on deformity. There are a few different ways to measure soft tissue laxity. Historically, many surgeons relied on their subjective assessment of soft tissue laxity and may have incorporated the use of lamina spreaders to tension the gaps [19]. With improved instrumentation, several companies have produced manual devices that allow gaps to be quantified more precisely [20, 21]. Modern robotic systems allowing for dynamic stress evaluation of the compartments can be very useful as they are time-saving, more accurate, and allow the surgeon to assess soft tissue laxity throughout the knee range of motion, thereby receiving real-time objective information prior to bone resections [22]. Understanding each individual patient’s unique soft tissue laxity prior to bone resections is intuitively critical to achieving balanced post-resection gaps in the medial and lateral compartments while staying within defined goals for component alignment and rotation. Specifically, this may allow for more predictable patient-specific bone resections and limit the degree of soft tissue releases that need to be made.

One limitation of this study was its retrospective nature, which relied on a review of data from the robotic knee system, resulting in the inability to identify extraneous variables that may have affected soft tissue laxity output. Second, we did not have data about the degree of preoperative deformity as long-leg standing radiographs were not available for all patients, so we organized them categorically and not continuously. A third limitation was that resection of the posterior cruciate ligament was sacrificed by surgeon preference, which may have affected the consistency of the subsequent stress examination between posterior cruciate ligament-sacrificing and posterior cruciate ligament-sparing groups. A fourth limitation was that the amount of varus and valgus stress applied to each knee could vary between surgeons or on a case-to-case basis as this stress was applied manually by the surgeon. However, this has been shown to be highly reproducible across measurements by a single surgeon and across measurements made by multiple surgeons of varying experience, which implies that the amount of force applied during a knee stress examination is not a significant variable when assessing soft tissue laxity and compartment opening [13].

## Conclusion

In conclusion, due to the high variability in soft tissue laxity and its limited predictability based on preoperative deformity, a soft tissue assessment prior to bone resections and soft tissue releases should improve both measured-resection and gap-balancing TKA techniques. This could create a pathway for a “hybrid” or “patient-specific” balancing technique. Future work that explores soft tissue laxity variability based on precise preoperative deformity may help surgeons better predict bone resections and soft tissue releases to achieve balanced gaps. Importantly, these workflow changes might have a substantial impact on patient function and satisfaction.

## Data Availability

The datasets used and/or analyzed during the current study are available from the corresponding author on reasonable request.
